# The Role of Intestinal Flora in the Regulation of Bone Homeostasis

**DOI:** 10.3389/fcimb.2021.579323

**Published:** 2021-03-12

**Authors:** Chengxiang Li, Guofu Pi, Feng Li

**Affiliations:** Department of Orthopaedics, The First Affiliated Hospital of Zhengzhou University, Zhengzhou, China

**Keywords:** intestinal flora, bone homeostasis, bone formation, bone resorption, probiotics, prebiotics

## Abstract

Intestinal flora located within the intestinal tract comprises a large number of cells, which are referred to as the second gene pool of the human body and form a complex symbiotic relationship with the host. The knowledge of the complex interaction between the intestinal flora and various life activities of the host is a novel and rapidly expanding field. Recently, many studies are being conducted on the relationship between the intestinal flora and bone homeostasis and indicate that the intestinal flora can regulate bone homeostasis *via* the host immune, metabolic, and endocrine systems. What’s more, based on several clinical and preclinical pieces of evidence, changing the composition and function of the host intestinal flora through the application of probiotics, prebiotics, and fecal microbiota transplantation is being considered to be a potential novel target for the regulation of bone homeostasis. Here, we searched relevant literature and reviewed the role of the intestinal flora in the regulation of bone homeostasis and its modulating interventions.

## Introduction

The bone is an important component of the human locomotor system, which has functions of moving, supporting, and protecting the body. Besides, the bone has functions of producing human mesenchymal stem cells, hematopoietic progenitor cells and storing minerals. As a dynamic organ, the bone is undergoing continuous remodeling at all stages of human life. The maintenance of its functions requires a dynamic balance of bone formation and absorption, which is called bone homeostasis. The regulation of bone homeostasis mainly depends on the synergistic effect of osteoblasts and osteoclasts. Osteoblasts carry out the action of bone synthesis and formation, while osteoclasts participate in the decomposition and resorption of the bone. When bone formation is dominant, bone homeostasis is characterized by anabolism, otherwise it is catabolism (Karsenty and Wagner, 2002; Li L. et al., 2019). Existing research results indicate that the maintenance of bone homeostasis is regulated by multiple mechanisms of the host immune system, metabolic system, and endocrine system (Carmeliet et al., 2015; D’Amelio and Sassi, 2016; Halmos and Suba, 2019).

The intestinal flora is a large number and variety of microbial flora colonized in the intestinal tract of the host, including bacteria, fungi, Archaea, and viruses, which form a complex symbiotic relationship with the host. It is believed that the human intestinal flora is mainly obtained from the mother at birth and influenced by various factors such as prenatal events, mode of delivery, geographical location, dietary patterns, use of antibiotics, age, *etc* (Kau et al., 2011; Yatsunenko et al., 2012; Thursby and Juge, 2017). With the development of the Human Microbiome Project (HMP) and Metagenomics of the Human Intestinal Tract (MetaHIT), as well as the progress of advanced technologies such as 16S rRNA coding gene sequencing, the intestinal flora has become a research hotspot in many fields. More and more scholars regard it as a new multicellular “organ” that can interact with the host in many ways (Clarke et al., 2014). Prior studies confirmed that the intestinal flora is an important factor in maintaining the dynamic balance of various life activities of host, including intestinal physiology (Collins, 2014), nutrient metabolism (Flint et al., 2012), host growth (Sommer and Bäckhed, 2013), energy balance (Turnbaugh et al., 2006), metabolic function (Tremaroli and Bäckhed, 2012), immune function and inflammatory process (Belkaid and Hand, 2014) and brain nerve function (Kennedy et al., 2017), *etc*. Therefore, when the intestinal flora changed or was disordered, the dynamic balance of the host may be interrupted, leading to some pathologic conditions, which include obesity (Greiner and Bäckhed, 2011), inflammatory bowel disease (Kostic et al., 2014), type 1 and type 2 diabetes (Qin et al., 2012; Dedrick et al., 2020), colorectal cancer (Feng et al., 2015), neurodegenerative diseases (Petra et al., 2015), rheumatoid arthritis (Breban, 2016), and many other diseases. Despite the limited number of studies related to the effect of intestinal flora on bone homeostasis, recent studies found that there are interesting and complex potential relationships between them. The intestinal flora appears to be a significant contributor to bone health and disease (Pedersini et al., 2020). As a new target for regulating bone homeostasis, the intestinal flora has attracted great attention in many disciplines, such as orthopedics, immunology, endocrinology, and so on.

## Intestinal Flora Is Involved in the Regulation of Bone Homeostasis

As an emerging research direction, there is little direct clinical data linking the intestinal flora and bone homeostasis. In fact, more pieces of evidence are mainly obtained from preclinical animal model experiments.

Sjögren et al. (2012) demonstrated that the bone mineral density of 7-week-old female germ-free mice was increased compared with that of conventionally raised mice; in addition, the bone mass of distal femur and bone volume/tissue volume (BV/TV) of germ-free mice were increased, while the number of osteoclasts, osteoclast precursor (CD11b^+^/GRl^−^) cells and CD4^+^T cells was decreased significantly. Further study showed that after colonizing the normal intestinal flora into germ-free mice at the age of 3 weeks, the bone mass and frequency of osteoclasts, osteoclast precursor (CD11b^+^/GRl^−^) cells and CD4^+^T cells were normalized (Sjögren et al., 2012). This suggests that the absence of intestinal flora may lead to an increase in bone mineral density of germ-free mice. On the contrary, Schwarzer et al. (2016) found weaker bone development of 8-week-old male germ-free mice whose bone development indicators (femur length, cortical bone thickness, *etc*.) are significantly decreased. Besides the germ condition of mice, some other factors like genetic background, sex, and age of mice also have effects on such results. Fransen et al. 2015) confirmed that there is a great difference in the immune response to intestinal flora between C57BL/6J mice used by Sjögren and BALB/c mice used by Schwarzer. In addition, Yan et al. (2016) found that colonization of sexually mature germ-free mice with the intestinal flora acutely reduces bone mass, but in long-term colonized mice, an increase in bone formation and growth plate activity predominates, resulting in equalization of bone mass and increased longitudinal and radial bone growth.

At the same time, many studies on antibiotics confirmed that antibiotics have an effect on the composition of host intestinal flora, which can significantly affect host skeletal development. Cho et al. (2012) intervened postnatal weaned mice with four subtherapeutic antibiotic regimens (penicillin, vancomycin, penicillin plus vancomycin, and chlortetracycline) and found the bone mineral density of the experimental group was significantly higher than that of the control group (without antibiotic intervention). Further studies (Cox et al., 2014; Nobel et al., 2015) confirmed that the intervention of low-dose antibiotics can affect the skeletal development of weaned mice by regulating intestinal flora to change the metabolism and related gene expression of the host.

In addition, in the animal model experiments related to the study of postmenopausal osteoporosis, Li et al. (2016) observed that germ-free mice had no significant bone loss compared with conventionally raised mice in the leuprolide-induced hypogonadism mice model, indicating that germ-free mice could resist the bone resorption caused by sex steroid deficiency. Furthermore, Li conducted a further experiment and found that a twice-weekly treatment of sex steroid-deficient mice with the probiotics *Lactobacillus rhamnosus* GG (LGG) or the commercially available probiotic supplement VSL#3 reduces intestinal permeability and dampens intestinal and BM inflammation, which can completely protect against bone loss, while the protective effect was absent in sex steroid-deficient mice treated with non-probiotic strain of *E. coli* or mutant LGG (Li et al., 2016). This finding is also supported by previous studies (Sjögren et al., 2012; Britton et al., 2014; Ohlsson et al., 2014).

The above results suggest that the intestinal flora may participate in the regulation of bone homeostasis by affecting the host immune system, host metabolism, and endocrine environment. What’s more, probiotics can be used as a new therapeutic target to maintain bone homeostasis and bone health after antibiotics by regulating the composition of the host intestinal flora.

## Intestinal Flora Regulates Bone Homeostasis Through the Host Immune System

Prior study found that germ-free mice have an immature mucosal immune system, in the bone marrow of whom the number of CD4^+^T cells and osteoclasts, as well as the expression levels of tumor necrosis factor-α (TNF-α) and interleukin-6 (IL-6) is decreased, suggesting that the intestinal flora has a role in promoting the maturation of the host immune system (Sjögren et al., 2012). Further studies have confirmed that TNF-α can activate the receptor activator of nuclear factor kappa-B (NF-*κ*B) ligand (RANKL) signaling pathway, which can inhibit the differentiation of mesenchymal stem cells into osteoblasts, thus inhibit bone formation and promote bone loss (Kitaura et al., 2013). Studies have shown that there is a close interaction between the immune system and bone metabolism, which was called “osteoimmunology”, representing the role of immune cells or immune-related factors in the regulation of bone homeostasis. These results indicate that intestinal flora can regulate bone homeostasis through the host immune system.

### Intestinal Mucosal Barrier

As the first line of defense of host immunity and epidemic prevention, the intestinal mucosa has a certain barrier function. It not only plays a vital role in maintaining host health by the ability of digesting and absorbing nutrients, but also provides the necessary selective barrier for the host to prevent pathogens and their products and other harmful substances from being transferred to the blood circulation of the body from the external environment. The barrier of intestinal mucosa includes the physical barrier of the intestinal epithelium and the chemical barrier of the mucus layer. The intestinal epithelium is composed of continuous monolayer intestinal epithelial cells bound together by tight junction proteins, which allow substances to move from the mucosal side of the epithelium to the serous side through transcellular and paracellular pathways, determining the permeability of the intestinal mucosa, while the mucous layer is secreted by goblet cells located on the luminal surface of the epithelium, which is important in limiting the ability of intestinal bacteria and pathogens to enter the host cells (Chelakkot et al., 2018). The destruction of the intestinal mucosal barrier can affect the absorption of nutrients and promote the transfer of pathogens to the bloodstream, leading to systemic inflammation and metabolic diseases (Salvo Romero et al., 2015). The absorption of nutrients such as calcium and phosphorus in the intestinal tract and the systemic inflammatory response of the host have a direct effect on bone homeostasis, which will be discussed in detail later in this article.

**Figure 1:**
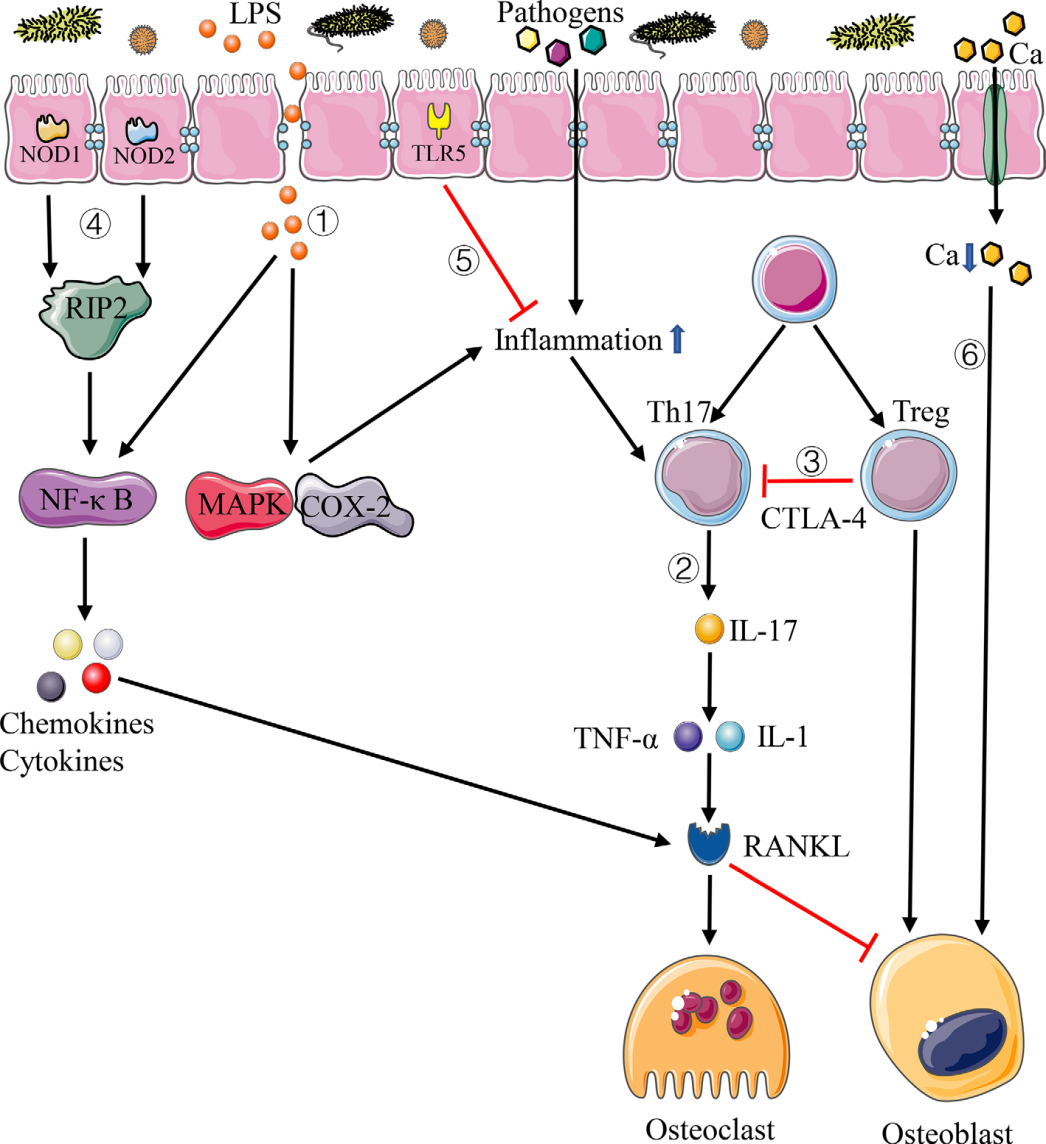
Pathways of immune system affecting bone homeostasis. 1) Disturbance of intestinal flora increases the permeability of intestinal mucosal barrier, letting more LPS and pathogens into the circulation system, which can lead to systemic inflammation. 2) Th17 cells produce IL-17, resulting in the increase of inflammatory cytokines such as TNF-α and IL-1, thus enhancing the expression of RANKL. 3) Treg cells inhibit the activation of T cells through CTLA-4-mediated pathway, thus inhibiting the differentiation of osteoclasts. 4) NOD1 and NOD2 bind peptidoglycans on the surface of bacteria and activate the NF-*κ*B pathway through RIP2, resulting in gene expression of chemokines and cytokines. 5) TLR5 binds with the flagellin of bacteria and inhibit the inflammation. 6) Disturbance of intestinal flora increases intestinal pH and decreases calcium absorption.

In the study of the intestinal mucosal barrier, Chen et al. (2018) demonstrated that the diversity and stability of the intestinal flora can enhance the integrity of the intestinal mucosal barrier and change the expression of inflammatory makers. Based on this, Zhou et al. (2017) proposed that the change of intestinal permeability can let intestinal bacterial antigens pass through the intestinal mucosal barrier, leading to biological dysregulation, increasing body inflammation, and then activating the T cells, resulting in enhanced expression of TNF-α. The increase of TNF-α can stimulate the formation of host osteoclasts and/or promote osteoblast apoptosis, thereby disrupting bone homeostasis and leading to bone resorption (Kobayashi et al., 2000). Meanwhile, Cani et al. (2007) illustrated that intestinal mucosal barrier dysfunction may lead to an increase level of serum lipopolysaccharide (LPS), which in turn increases intestinal mucosal permeability and leads to metabolic endotoxemia. Moreover, Huo and Itoh et al (Itoh et al., 2003; Hou et al., 2013). confirmed that LPS can promote the differentiation and survival of osteoclasts *in vitro* and then increases the bone loss by enhancing the MAPK pathway and the expression of cyclooxygenase-2 (COX-2) in RAW264.7 cells and Toll-like receptor 4 (TLR4) in different ways (**Figure 1**). In addition, the germ-free mice study on postmenopausal osteoporosis by Li mentioned above also shows that LGG and probiotic supplement VSL#3 can prevent bone loss caused by estrogen deficiency through reducing intestinal permeability and intestinal inflammation (Li et al., 2016). Therefore, the intestinal flora can participate in the regulation of host bone homeostasis through the effect of the intestinal mucosal barrier.

### Helper T Cells and Regulatory T Cells

Cellular immune response mediated by T cells takes a significant role in immunity. The intestinal flora is involved in the induction of cytokine production and lymphocyte development, especially the Th17 cells and Treg cells (Atarashi et al., 2011; Lee et al., 2011). As a subset of CD4^+^T cells, Thl7 cells play vital roles in maintaining mucosal barrier and preventing bacteria pathogen from invading the intestinal tract. Th17 cells activated by intestinal inflammation can migrate to the bone matrix and produce IL-17 to enhance local inflammation, resulting in the increase of inflammatory cytokines such as TNF-α and IL-1, thus enhancing the expression of RANKL and activating osteoclast precursor cells to promote osteoclast differentiation (Sato et al., 2006) (**Figure 1**). It has also been proved that the elimination of IL-17 or the use of anti-IL-17 antibodies can prevent bone loss (DeSelm et al., 2012; Tyagi et al., 2014). Treg cells exist stably in the intestinal mucosa and have a measurable influence on intestinal and systemic immune system. Among them, CD4^+^CD25^+^Foxp3^+^Treg cells can inhibit T cell activation through the pathway mediated by cytotoxic T lymphocyte associated protein 4 (CTLA-4), which can reduce the expression of RANKL and other cytokines and inhibit the differentiation of osteoclasts so as to alleviate the process of bone resorption and promote bone formation (Kong et al., 1999) (**Figure 1**). In the study of estrogen deficiency osteoporosis, Zaiss et al. (2010) confirmed that estrogen can directly increase the relative number of Treg cells, thus preventing the occurrence of bone loss induced by ovariectomy in mice.

### Nucleotide-Binding Oligomerization Domain 1, NOD2, and Toll-Like Receptor 5

The relationship between the intestinal flora and bone homeostasis is also reflected through non-specific immunity mediated by NOD1, NOD2, and TLR5. NOD1 and NOD2 are ubiquitous intracellular receptors of pathogen-associated molecular patterns (PAMPs), mainly expressed on epithelial cells and immune cells, which can bind peptidoglycans on the surface of bacteria and activate the NF-*κ*B pathway through receptor-interacting protein 2 (RIP2), resulting in gene expression of chemokines and cytokines, and play a key role in the effect of intestinal flora on bone (Li L. et al., 2019) (**Figure 1**). Ohlsson et al. (2017) demonstrated that neither the expression of TNF-a and RANKL nor the change of bone density was affected by the change of intestinal flora in mice after knocking out NOD1 and NOD2. The above results indicate that the change of bone mass in germ-free mice depends on the effect of these two proteins.

TLR5 is a non-specific immune receptor of flagellin, one of the major proteins of bacteria, and it is expressed on both immune cells and non-immune cells. TLR5 knockout mice would cause changes in the intestinal flora due to defects in the immune system, resulting in an increase in the number of Proteobacteria and flagellated bacteria in mice intestinal tract, which promotes a significant increase in inflammation and metabolic activities in the body (Cullender et al., 2013) (**Figure 1**). What’s more, the existence of this immunodeficiency can influence the interaction between the host immune system and the body’s skeleton. In the study of intestinal flora and bone strength, Guss et al. (2017) found that the bone phenotype of TLR5 knockout mice was significantly different from that of wild-type mice, with larger bone cross-sectional area and moment of inertia and lower whole bone strength. Besides, Guss also found that the use of antibiotics can cause a greater decrease in the whole bone bending strength of the femur in TLR5 knockout mice compared with wild-type mice (Guss et al., 2017).

### Wnt Signaling Pathway

Wnt signaling pathway is a highly conserved signaling pathway widely existing in animals. It acts major roles in many fields, such as animal embryonic development, tissue differentiation, tumorigenesis, bone development, and metabolism. The activation of Wnt signaling pathway causes high bone mass, while inactivation leads to low bone mass and early osteoporosis (Kobayashi et al., 2016). Among the 19 Wnt proteins discovered in mammalian Wnt protein family, Wnt1, Wnt3a, Wnt4, Wnt5a, Wnt7b, Wnt10b, and Wnt16 have been proved to regulate the process of bone metabolism in different ways (Lerner and Ohlsson, 2015). The canonical Wnt signaling pathways, Wnt/*β*-catenin signaling pathway, can regulate the expression of osteoblast target genes by enriching *β*-catenin in cells, promoting bone mineralization and differentiation of osteoblasts and chondrocytes and prolonging the survival time of osteoblasts to affect the bone resorption (Zhong et al., 2012). Additionally, it can also affect bone resorption by inhibiting the coupling between osteoblasts and osteoclasts and regulating the expression of osteoprotegerin (OPG) and RANKL (Zhong et al., 2012). From infancy to adulthood, the function of osteoblasts is regulated by Wnt/*β*-catenin signaling in almost every aspect. The relationship between this signaling pathway and the intestinal flora has become a research hotspot in recent years. Tyagi et al. (2018) demonstrated that LGG and butyrate can induce the expansion of intestinal and bone marrow (BM) Treg cells, which can interact with BM CD8^+^ T cells, resulting in increased secretion of Wnt10b, a bone anabolic Wnt ligand, thereby activating Wnt signaling in osteoblastic cells and indirectly stimulating bone formation. Meanwhile, Yang et al. (2013) found that intestinal microorganisms can polarize colonic macrophages into M1 state, thus producing endogenous inflammatory cytokines, causing microbial-induced bystander effect (MIBE), which can activate Wnt/*β*-catenin signaling pathway with the help of TNF-α. Based on the discussion above, we believe that the intestinal flora can indirectly regulate host bone homeostasis through the Wnt signaling pathway.

## Intestinal Flora Regulates Bone Homeostasis Through Host Metabolic System

There is an interactive relationship between the intestinal flora and host metabolic system. The host can affect the composition of the intestinal flora through dietary changes (David et al., 2014), while the disorder of the intestinal flora can affect host metabolism and cause many metabolic diseases such as obesity, diabetes, and so on. Prior studies confirmed that the intestinal flora can affect host metabolism and bone homeostasis in many ways, which will be discussed thoroughly in the following subsections.

### Absorption of Calcium and Vitamin D

Calcium is the main mineral of the bone; keeping the positive calcium balance is important to maintain the bone homeostasis. Calcium can be absorbed by the intestinal cells through active transcellular pathway and passive paracellular diffusion, then deposited in the bone in the form of hydroxyapatite. Vitamin D can affect calcium absorption; in addition, vitamin D and its metabolites can maintain the homeostasis of the intestinal flora, improve calcium balance, and promote mineral deposition in the bone matrix. Deficiency of calcium or vitamin D could result in severe abnormalities of bone metabolism (Xu et al., 2017). In 2003, Stotzer et al. (2003) pointed that patients with small intestinal bacterial overgrowth syndrome were not only with low bone mineral density, osteomalacia, and increased osteoclast activity, but also with the characteristic of nutritional deficiency which was caused by the nutrient consumption of the intestinal flora. Therefore, it is suspected that the insufficient absorption of nutrients such as calcium and vitamin D caused by intestinal flora disorder contributes to the skeletal manifestation of the syndrome. Also, the process by which the intestinal flora regulates bone homeostasis by affecting the absorption of calcium and vitamin D has also been supported by recent studies.

In a study of *Lactobacillus reuteri*, it has been shown that the use of this intestinal probiotic in healthy subjects can increase serum levels of 1,25-dihydroxyvitamin D3 [1,25-(OH)_2_D3], which promotes calcium absorption and is beneficial to bone health (Jones et al., 2013). Meanwhile, Rodrigues et al. (2012) demonstrated that Bifidobacteria can produce short-chain fatty acid (SCFA) that can reduce intestinal pH, which is conducive to the dissolution of minerals and the transportation of calcium ions to cells through the paracellular pathway and then regulate bone mineral density. Studies on adolescents also confirmed that the intestinal flora can ferment soluble corn fiber (SCF) to SCFA, thus affecting intestinal pH, reducing the formation of calcium phosphate, and increasing calcium absorption (Whisner et al., 2016). In addition, studies on bone metabolism in postmenopausal women also found that *Lactobacillus helveticus* can reduce the level of parathyroid hormone (PTH), increase the concentration of serum calcium, and promote calcium absorption (Narva et al., 2004).

### Short-Chain Fatty Acid

**Figure 2:**
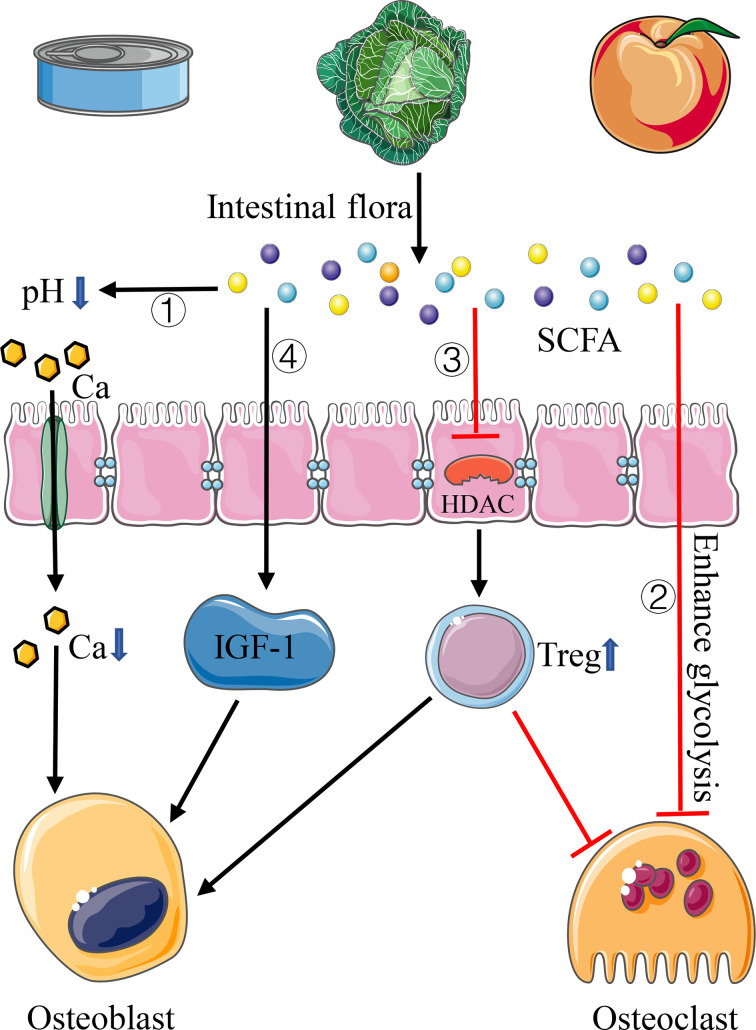
Pathways of SCFA affecting bone homeostasis. 1) SCFA reduces intestinal pH and increases calcium absorption. 2) SCFA inhibits the differentiation of osteoclasts by increasing the occurrence of intracellular glycolysis. 3) SCFA inhibits the activity of HDAC, thus increasing the differentiation of Treg cells. 4) SCFA can induce the increase of IGF-I.

SCFA is produced by the fermentation of complex indigestible carbohydrates in the diet through the intestinal flora, including acetate, propionate, and butyrate (**Figure 2**). Acetate and propionate can also be produced by intestinal bacteria when they break down amino acids and lactic acid. Recently, many studies confirmed that the intestinal flora plays an important role in regulating bone homeostasis by SCFA through diverse pathways, except that SCFA can regulate bone homeostasis by reducing intestinal pH, changing mineral solubility, and increasing calcium transport into cells in the intracellular pathway (**Figure 2**). Cantley et al. (2011) illustrated that butyrate and propionate can directly inhibit the differentiation of human osteoclasts by increasing the occurrence of intracellular glycolysis at the early stage of osteoclast differentiation (**Figure 2**). Other scholars found that butyrate and propionate can regulate gene expression by inhibiting the activity of histone deacetylase (HDAC), thus increasing the differentiation ability of Treg cells and affecting the balance between pro- and anti-inflammatory cells (Arpaia et al., 2013; Smith et al., 2013) (**Figure 2**). As mentioned in the section *Helper T Cells and Regulatory T Cells* above, Treg cells can inhibit osteoclast differentiation, prevent bone resorption, and promote bone formation. Lucas confirmed the effect of SCFA on osteoclasts in mice. After treating osteoporotic mice with butyrate and propionate, they found that the number of osteoclasts and bone resorption markers decreased significantly (Lucas et al., 2018). Additional studies showed that SCFA can induce the increase of insulin-like growth factor-I (IGF-I), which is important in promoting bone formation (Yan et al., 2016) (**Figure 2**). In addition, as we mentioned above, Tyagi et al. (2018) demonstrated that the application of butyrate in mice model can induce T cells to produce Wnt10b protein, which can activate the Wnt signaling pathway in osteoblasts, then promoting osteoblast production and reducing osteoblast apoptosis, thus increasing bone mass in mice.

### Bile Acids

**Figure 3:**
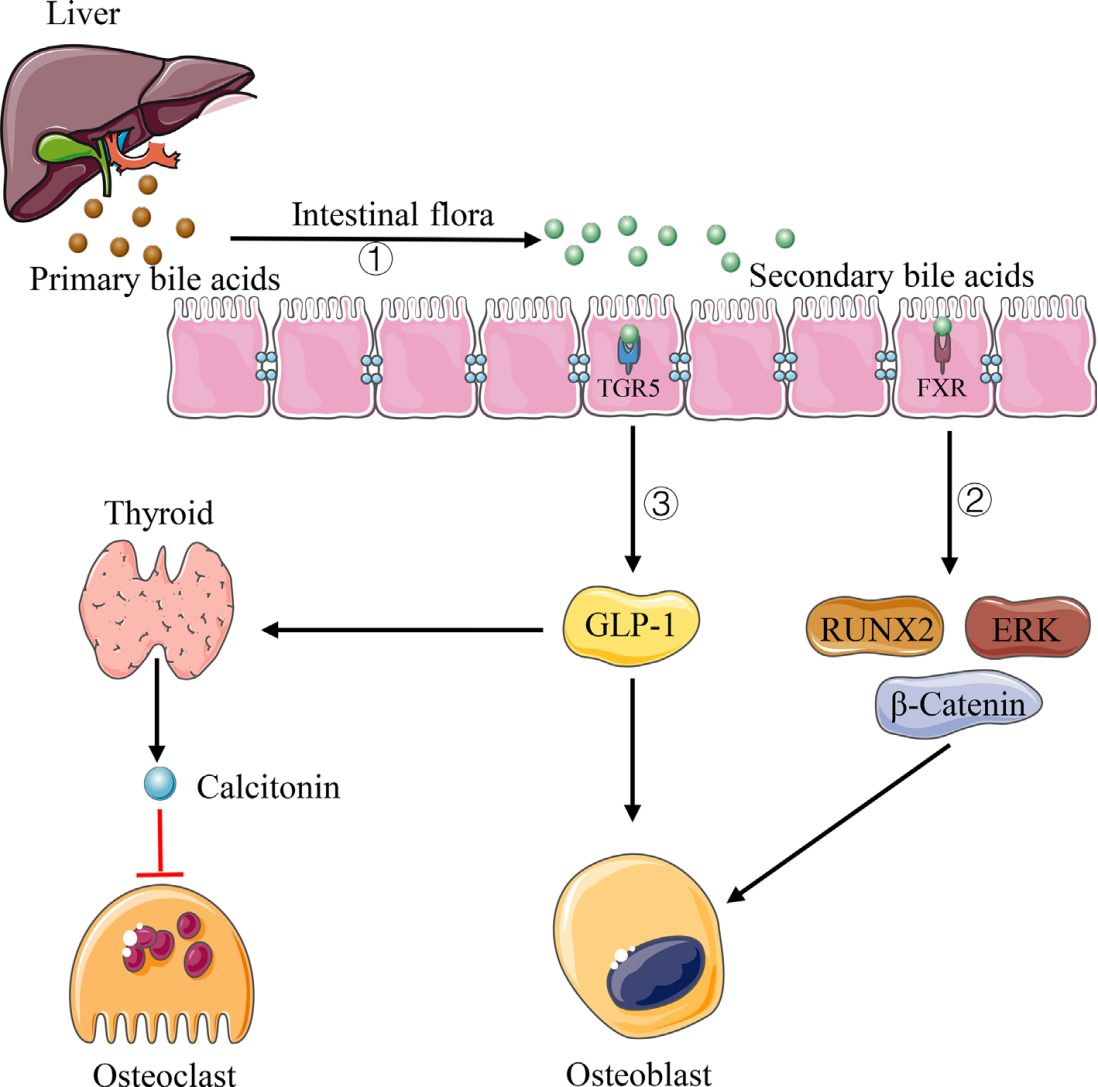
Pathways of bile acids affecting bone homeostasis. 1) Intestinal flora can convert primary bile acids into secondary bile acids. 2) Some types of secondary bile acids can up-regulate the expression of Runx2 and enhance ERK and *β*-catenin signaling *via* FXR. 3) Some types of secondary bile acids increase GLP-1 production *via* TFR5, causing thyroid cells to secrete calcitonin through paracrine.

Bile acids are metabolic molecules produced by the liver, which can be secreted into the intestinal tract and participate in the absorption of dietary fat. As an essential participant in bile acid metabolism, the intestinal flora can bioconvert primary bile acids into unconjugated and secondary bile acids through de-conjugation and dehydroxylation reactions (Singh et al., 2019) (**Figure 3**). There is growing evidence that bile acids can regulate bone dynamic balance through various signals of osteoblasts and osteoclasts. Cho et al. (2013) showed that bile acids can activate farnesol X receptor (FXR) signaling *in vitro* and significantly increase osteoblast mineralization by up-regulating the expression of Runx2 and enhancing extracellular signal-regulated kinase (ERK) and *β*-catenin signaling (**Figure 3**). In addition, when the intestinal flora converts primary bile acids into secondary bile acids, the secondary bile acids, as an agonist of G protein-coupled bile acid receptor (TGR5), can activate TGR5 to increase the production of glucagon-like peptide-1 (GLP-1), which can cause thyroid cells to secrete calcitonin through paracrine, thus inhibiting bone resorption (Sandoval and D’Alessio, 2015) (**Figure 3**). GLP-1 can also stimulate the proliferation and differentiation of osteoblasts and promote bone formation (Sandoval and D’Alessio, 2015). What’s more, as one of the secondary bile acids and vitamin D receptor ligand, lithocholic acid (LCA) can also affect bone homeostasis. Prior studies indicated that the excessive increase of LCA *in vivo* not only can destroy the activity of osteoblast mitochondria and reduce the vitality of osteoblasts, but also decrease the effect of vitamin D in osteoblasts and inhibit the expression of osteocalcin and RANKL gene (Ruiz-Gaspà et al., 2010).

### Lipopolysaccharide

LPS is a main component of the cell wall of Gram-negative bacteria, which can stimulate inflammation in the host by the activation of transforming growth factor (TGF) and Toll-like receptor 4 (TLR4) (Manco et al., 2010). It will cause more LPS to enter the circulatory system when the host’s intestinal flora is disordered and the number of Gram-negative bacteria increases, thus leading to metabolic dysfunction and inflammation. LPS also has a certain role in the regulation of bone homeostasis. In the mice implanted with LPS to simulate a chronic inflammation model, Smith et al. (2006) found a loss of femoral bone and a decrease of bone mineral density; besides, the volume of the trabecular bone in the proximal metaphysis of the tibia in the high-dose LPS-treated group tended to decrease, and the expression of interleukin-1 (IL-1), cyclooxygenase-2 (COX-2) as well as tumor necrosis factor (TNF) was up-regulated. Chang et al. (2009) also confirmed that LPS can activate NF-*κ*B signaling pathway, and thereby lead to the production of inflammatory cytokines such as IL-1, TNF-α, and prostaglandin E2, which can stimulate the activation and differentiation of osteoclasts, resulting in the increase of bone resorption.

## Intestinal Flora Regulates Bone Homeostasis Through the Host Endocrine System

As a new “organ” of the human body, the intestinal flora can regulate bone homeostasis through interaction with the immune system and metabolic system. Furthermore, the intestinal flora has been proved to be closely related to the endocrine system that can regulate bone homeostasis by affecting the secretion level and activity of related hormones in the body.

### Sex Hormones

Sex hormones are steroid hormones mainly produced by gonads, which act a major role in the growth and development of the body. A large number of studies have also confirmed that sex hormones have a regulatory effect on bone homeostasis. Due to the expressions of androgen receptor (AR) on the surface of osteoblasts and estrogen receptor (ER) on the surface of osteoblasts, osteoclasts and osteocytes, some researchers believe that sex hormones can regulate bone homeostasis through the direct effect on osteoblasts and osteoclasts. Nakamura et al. (2007) confirmed that estrogen can directly promote osteoclast apoptosis and protect the body from osteoclast formation by binding with ER and inducing the activation of Fas/FasL pathway in osteoclasts. The experiment of Kousteni et al. (2001) not only proved that estrogen could directly induce osteoclast apoptosis and inhibit osteoblast apoptosis, but also proved the protective effect of androgen on bone loss by AR. Moreover, sex hormones can also regulate bone homeostasis through the immune response in the host. It is proved that estrogen deficiency in the host can promote the proliferation and differentiation of T cells including Th17 by increasing the antigen presentation function of dendritic cells (DCs) and macrophages, and inducing the increase of interferon-*γ* (IFN-*γ*), IL-7 and the decrease of transforming growth factor-*β* (TGF-*β*), then producing large quantities of TNF-α, which can promote osteoclast differentiation by directly acting on osteoclast progenitor cells and stimulate osteoclast formation indirectly by activating host CD40/CD40L system to promote macrophage differentiation and stromal cells expressing Macrophage Colony-stimulating factor (M-CSF) and RANKL (Cenci et al., 2003; Pacifici, 2012; Tyagi et al., 2012).

In addition, a large number of studies have verified that the intestinal flora and sex hormones can interact with each other. As we mentioned in *Intestinal Flora Is Involved in the Regulation of Bone Homeostasis*, Li et al. (2016) proved that the intestinal flora plays a vital role in bone loss caused by sex hormone deficiency. Ridlon et al. (2013) proposed that the intestinal symbiotic bacterium *Clostridium sinensis* can convert glucocorticoids into androgens under the action of hydroxysteroid hydrolase and other enzymes. Moreover, Plottel et al. (2011) demonstrated that bacteria with estrobolome gene in the intestinal flora can affect the metabolism of estrogen by accelerating the early dissociation and hydroxylation of estrogen in the intestinal tract, thus enhancing the reabsorption of estrogen and increasing the number of free estrogens in the hepatoenteral circulation. Under the activation of the liver, free estrogens can play a biological role. What’s more, in an experiment that transplanted the intestinal flora of male mice into the intestinal tract of female mice, it was found that the transplantation of flora could raise the level of serum testosterone in female mice, which is the principal component of androgen (Markle et al., 2013). These results suggest that the intestinal flora can affect the level of sex hormones in the host, thereby modulating bone homeostasis.

### Insulin-Like Growth Factor-1

**Figure 4:**
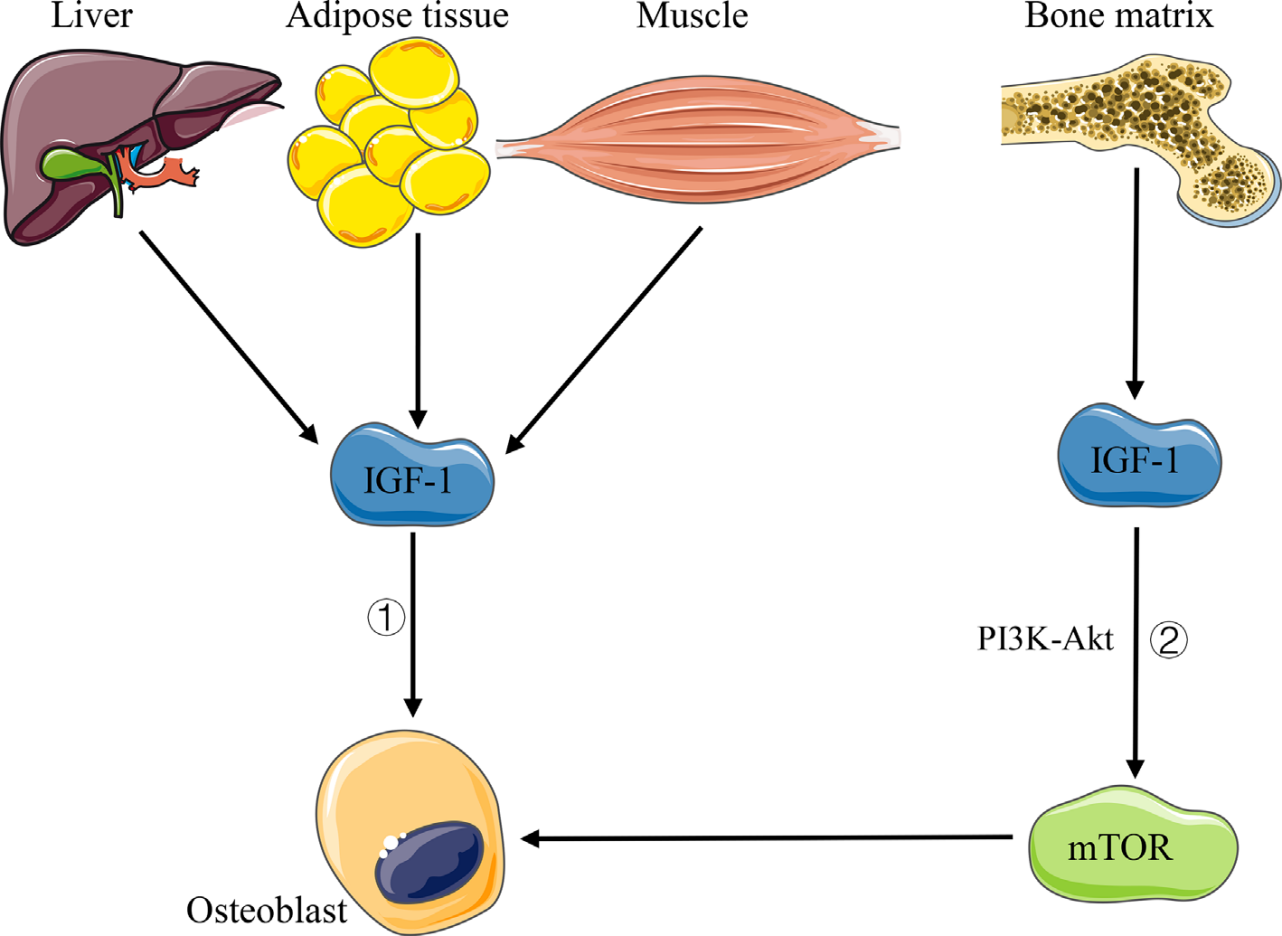
Pathways of IGF-1 affecting bone homeostasis. 1) IGF-1 acts directly on osteoblasts. 2) IGF-1 released by bone matrix activates mTOR signaling through PI3K–Akt pathway and induces the differentiation of osteoblasts.

IGF-1 is a hormone known to have an effect on bone growth. The liver is the main contributor (about 75%) of the body’s IGF-1 circulating pool, while other tissues, such as fat and muscle, contribute the remaining 25% (Yakar and Isaksson, 2016). Importantly, almost all tissues of the body produce IGF-1, which takes effects in an autocrine or paracrine manner (Yakar and Isaksson, 2016). Studies showed that osteoblasts are the main regulatory targets of IGF-1 in bone homeostasis, which can directly act on the IGF-1 receptor on osteoblasts, thus inducing the recruitment and differentiation of osteoblasts, increasing the production of type 1 collagen and the activity of alkaline phosphatase, and then promoting bone formation (Wang et al., 2007) (**Figure 4**). Moreover, Xian et al. (2012) demonstrated that in the process of bone remodeling, IGF-1 released upon osteoclast-dependent degradation of the bone matrix can activate the mTOR signaling pathway through PI3K–Akt pathway and induce the differentiation of bone marrow mesenchymal stem cells (MSCs) into osteoblasts (**Figure 4**). Another study on bone development showed that IGF-I-deficient mice express a significant decrease in chondrocyte proliferation and an increase in apoptosis, as well as abnormal chondrocyte differentiation, resulting in the inhibition of bone linear growth and a dramatic decrease of spinal bone density (Wang et al., 2006). In addition, the change of the intestinal flora can affect the level of serum IGF-1. Yan et al. (2016) found a decreased level of serum IGF-1 in germ-free mice and antibiotic-treated wild-type mice, but an increased level of IGF-1 and bone formation after intestinal flora colonization in germ-free mice or supplementation of SCFA in antibiotic-treated mice. Storelli et al. (2011) observed that microbiota-dependent activation of the TOR pathway in adipose tissue (fat bodies) and in a neuroendocrine organ (the prothoracic gland) contributes to the increased production of the insulin-like/IGF1-like peptides, with systemic effects. Therefore, we believe that the intestinal flora can regulate bone homeostasis by affecting the level of host IGF-1.

### 5-Hydroxytryptamine and Brain–Gut–Bone Axis

**Figure 5:**
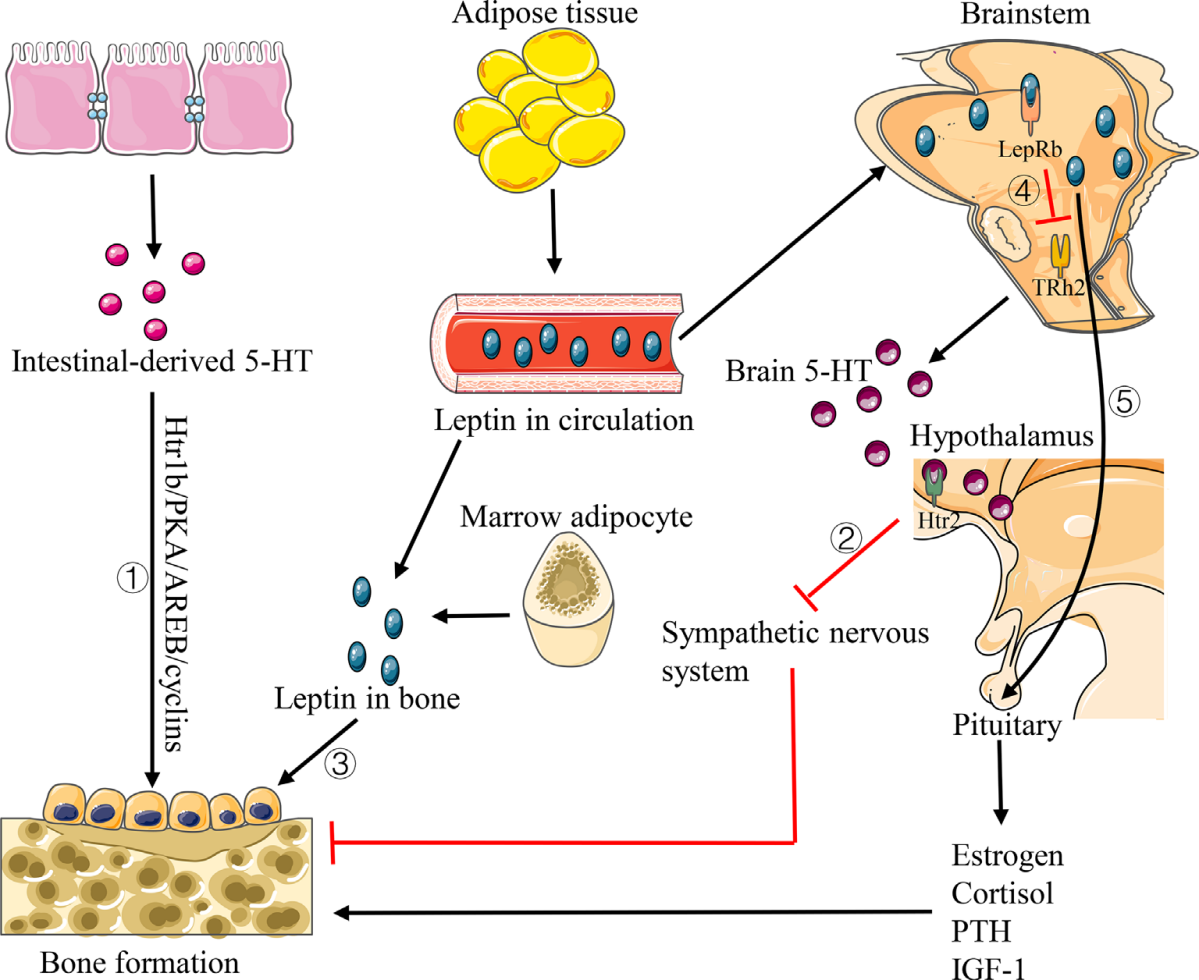
Pathways of 5-HT and leptin affecting bone homeostasis. 1) Intestinal-derived 5-HT activates Htr1b on osteoblast progenitor cells and regulates its proliferation *via* Htr1b/PKA/CREB/cyclins signaling pathway. 2) Brain 5-HT promotes bone formation by acting on Htr2c to reduce the sympathetic activity. 3) Leptin has a direct anabolic effect on osteoblast lines. 4) Leptin decreases the production of brain 5-HT. 5) Leptin increases the levels of estrogen, cortisol, IGF-1 and PTH, thus promoting bone formation.

As a neurotransmitter, 5-HT takes a dual role in the bone development regulation. Ducy and Karsenty et al. (2010) proved that 5-HT produced in the peripheral circulation has a negative regulatory effect on bone metabolism, while it can positively regulate bone metabolism by promoting bone formation and inhibiting bone resorption when it is produced by the central nervous system. Two studies conducted by Yadav et al. (2008) and Yadav et al. (2009). proved that the intestinal-derived 5-HT can activate 5-HT1b receptor (Htr1b) on osteoblast progenitor cells, then inhibiting the proliferation of osteoblasts through Htr1b/PKA/CREB/cyclin signaling pathway, while the brain-derived 5-HT has a beneficial effect on bone metabolism, which can reduce sympathetic nerve activity by acting on 5-HT2c receptor (Htr2c) expressed in the ventral nucleus of the hypothalamus (**Figure 5**). Animal experiments in ovariectomized mice also demonstrate that drugs inhibiting the synthesis of intestinal-derived 5HT can prevent osteoporosis by increasing bone formation (Yadav et al., 2010). In recent years, many studies suggested that the intestinal flora takes a role in regulating the level of 5-HT in body. Sjögren et al. (2012) observed a decrease in 5-HT level and an increase in bone volume fraction (BV/TV) in germ-free mice. Except for strengthening the result that there is a decrease in 5-HT level in germ-free mice, Yano et al. (2015) also confirmed that indigenous spore-producing anaerobic bacteria from normal mice and human intestinal tract could promote the biosynthesis of 5-HT from colonic enterochromaffin cells (ECs), and after colonizing the bacteria in germ-free mice, the level of 5-HT in the serum and colon could be restored. Reigstad et al. (2015) illustrated that the intestinal flora can promote the production of 5-HT in the colon by the effect of SCFA on intestinal chromaffin cells. Thus, we can infer that the intestinal flora would regulate bone homeostasis by affecting the level of 5-HT in the host.

Brain-derived 5-HT can regulate bone homeostasis by affecting the host nervous system, which suggests that intestinal flora may regulate bone homeostasis with the nervous system through the action of some neurotransmitters. Consequently, some scholars have proposed a “brain–gut–bone axis” regulation system. However, there is little research in this field, and we still need further research to reveal the possible mechanism. This regulatory system would be used as a new direction for the treatment of bone diseases in the future.

### Leptin

Leptin is a multifunctional protein hormone mainly secreted by the adipose tissue, which has an important effect on human energy metabolism. In the study of bone microstructure of leptin deficiency mice, Hamrick et al. (2004) found that there are decreases in cortical thickness, trabecular volume, bone mineralization, and bone mineral density in the peripheral femur of mice, while an increase in the lumbar vertebrae of the axial bone. Leptin plays a dual role in regulating bone homeostasis in different approaches. Gordeladze et al. (2002) demonstrated that leptin has a direct synthetic and metabolic effect on osteoblast lines. It can stimulate the differentiation of human bone marrow stromal cells into osteoblasts and increase the synthesis of bone matrix type I collagen and osteocalcin to promote bone formation (**Figure 5**). On the contrary, Ducy et al. (2000) found that the trabecular volume of the vertebrae decreased significantly to the level below wild-type by intracerebroventricular injection of leptin in both leptin deficient and wild-type mice, indicating that leptin can inhibit bone formation through the central nervous system (**Figure 5**). In addition, leptin has also been shown to regulate bone homeostasis indirectly by affecting the levels of estrogen, cortisol, IGF-1, and parathyroid hormone (PTH) (Upadhyay et al., 2015) (**Figure 5**). What’s more, some other evidence suggests that leptin levels can be affected by the intestinal flora. In the study of intestinal flora and serum leptin in mice with different nutritional status, Queipo-Ortuño et al. (2013) demonstrated that the number of *Bifidobacterium* and *Lactobacillus* in each experimental group was positively correlated with the level of serum leptin, while the number of *Clostridium*, *Bacteroides* and *Prevotella* was negatively correlated with the level of serum leptin. Park et al. (2013) also confirmed that eating *Lactobacillus curvatus* and *Lactobacillus plantarum* can reduce the level of leptin in mice. Additional studies showed that *Bifidobacterium pseudocatenulatum* CECT7756 can reverse hyperleptinemia and restore leptin signals in obese mice (Agusti et al., 2018). In addition, inulin, as a kind of prebiotics, has also been proved to alter the regulatory effect of leptin on the body by affecting the intestinal flora (Song et al., 2019).

### PTH

PTH, an important regulator of bone homeostasis, is a peptide hormone synthesized in the chief cells of the parathyroid glands. PTH can promote both bone resorption and formation, mainly depending on whether target cells are exposed to it continuously or intermittently. Continuous exposure to PTH (cPTH) results in catabolic effects on the bone, while intermittent exposure of PTH (iPTH) results in osteoanabolic effects (Locklin et al., 2003). A key mechanism whereby cPTH induces bone resorption is the OPG-RANKL–RANK pathway. cPTH can increase mRNA encoding for RANKL and decrease mRNA encoding for OPG in primary murine osteoblasts and in the bone, leading to an increased RANKL/OPG ratio and consequently, enhanced the differentiation/recruitment of osteoclasts and bone resorption (Ma et al., 2001). In addition, T cells are also targeted by PTH and play a role in the bone catabolic activity of cPTH by up-regulating the capacity of osteocytes and osteoblasts to release RANKL in response to PTH (Li et al., 2015; Li J.Y. et al., 2019), while the bone formation obtained with iPTH is mainly due to the activation of Wnt signaling pathway, which leads to the enhancement of osteoblast activity and bone formation. The mechanism by which iPTH activates Wnt signaling includes blunted osteocytic production of the Wnt inhibitor sclerostin (Bellido et al., 2005), decreased production by osteoblasts of the Wnt inhibitor Dickkopf-1 (Guo et al., 2010), and increased in the number of Treg cells (Yu et al., 2018).

With the deepening of microbiota research, recent studies have confirmed that the intestinal flora is pivotal for the bone catabolic and anabolic activity of iPTH. Yu et al. (2020) found that cPTH treatment does not induce bone loss in conventional mice treated with antibiotics or in germ-free mice, but only in mice whose microbiota was enriched by the Th17 cell-inducing taxa segmented filamentous bacteria (SFB), thus implicating the intestinal flora in the skeletal response to PTH. The authors went on to show that the SFB^+^ microbiota enabled PTH to expand intestinal TNF-producing T cells and Th17 cells and increase their S1P-receptor-1 mediated egress from the intestine and recruitment to the BM, resulting in bone loss, which indicates that the intestinal flora is a required determinant of the skeletal effects of cPTH (Yu et al., 2020). Similarly, Li et al. (2020) illustrated that iPTH treatment did not induce trabecular bone anabolism in germ-free mice and antibiotic-treated conventional mice, indicating that the intestinal flora is required for the anabolic activity of iPTH in the trabecular bone. To investigate the role of the intestinal flora in the skeletal response to iPTH, further experiments were carried out by the authors. They found that butyrate produced by the intestinal flora was required for PTH to expand Tregs and to increase Wnt10b expression by CD8+ T cells, which activated Wnt-dependent bone formation (Li et al., 2020). All these studies demonstrate the importance of the intestinal flora in allowing PTH to exert its effects on bone resorption and formation.

## The Possibility of Regulating the Composition and Function of Intestinal Flora for the Maintenance of Bone Homeostasis

For decades, low-dose antibiotics have been used as agents to regulate the body’s inflammatory response and promote bone formation of the host (Cromwell, 2002). However, with the widespread use of antibiotics, scholars have gradually discovered the residues of antibiotics in the host and the continuous production of drug-resistant strains, which force people to seek better treatments. In addition, the continuous exploration of the intestinal flora enables us to realize that the intestinal flora is an important factor to maintain the dynamic balance of various life activities, including host bone homeostasis. Therefore, scholars have been widely concerned with changing the composition and function of the intestinal flora to maintain bone homeostasis.

### Application of Probiotics

Probiotics are living symbiotic microorganisms, which are beneficial to host health when sufficient quantities are given. Recent studies have shown that probiotics can change the composition of the intestinal flora through their own colonization and proliferation in the intestinal tract, as well as the ability to compete with other bacterial strains, thus changing the intestinal flora, intestinal mucosal barrier, and host immune function to maintain bone homeostasis (McCabe and Parameswaran, 2018). As mentioned in the section *Intestinal Flora Is Involved in the Regulation of Bone Homeostasis*, probiotic LGG and probiotic supplement VSL#3 can reduce intestinal permeability, dampen intestinal inflammation, and prevent bone loss caused by estrogen deficiency (Li et al., 2016). Ohlsson et al. (2014) confirmed that probiotics can also protect mice from bone loss induced by ovariectomy through reducing the expression of the two inflammatory cytokines (TNF-α and IL-1β) and increasing the expression of OPG in the bone of ovariectomized mice. Another study showed that oral *Lactobacillus reuteri* can reduce intestinal inflammation and improve bone density in male mice with intact gonads (McCabe et al., 2013). Apart from these, probiotics have been suggested as an adjuvant treatment for focal bone loss such as periodontitis and alveolar erosion. Different *Lactobacillus* strains have been shown to reduce the number of osteoclasts and prevent alveolar erosion and tooth movement in rats and mice (Ricoldi et al., 2017). This conclusion has also been confirmed in a human study (Gruner et al., 2016). The above studies show that maintaining bone homeostasis by probiotics is an effective way; nevertheless, the efficacy can be affected by the different compositions of intestinal flora among different species and different hosts (Kiousi et al., 2019). Consequently, the best efficacy of probiotics can be guaranteed by considering the specificity of strain and host, which would be a sensible research direction in the future.

### Application of Prebiotics

As a kind of fermentable food ingredient that cannot be digested by the host, prebiotics can be used as metabolic substrates to stimulate the growth and activity of one or more bacteria in the intestinal flora; in this process, the metabolites produced by the intestinal flora can be used by the host (Roberfroid et al., 2010). Prebiotics mainly include a variety of indigestible oligosaccharides, the most common of which are galactooligosaccharides (GOS), fructooligosaccharides (FOS), xylooligosaccharide (XOS), inulin, and lactulose (Locantore et al., 2020). Prebiotics also naturally exist in vegetables, fruits, and other high-fiber foods (Locantore et al., 2020). SCFA is one of the fermentation products of prebiotics through the intestinal flora, which has been proved to play an important role in maintaining host bone homeostasis. Weaver et al. (2015) confirmed that the application of prebiotics can change the composition of the intestinal flora; for instance, FOS and GOS can increase the proportion of bifidobacteria in the intestinal flora and regulate bone homeostasis by affecting the production of SCFA. In an experiment on ovariectomized rats, inulin intake and FOS can cause an increase in the absorption of host calcium and bone mineral density, which can effectively prevent estrogen deficiency-induced bone loss (Zafar et al., 2004). In addition, a human controlled feeding study of adolescent women confirmed that the calcium absorption of the treatment group was increased, as well as the number of *Parabacteroides* and clostridium in the intestinal flora, which was associated with SCF fermentation and the production of SCFA (Whisner et al., 2016). Moreover, in a study about the effects of prebiotics on the bone of adolescents, Abrams et al. (2005) proved that the long-term application of inulin-type Fructan can promote bone mineralization in adolescent growth and increase bone density of the body. Meanwhile, Slevin et al. (2014) found that after taking short-chain Fructo-oligosaccharides (ScFOSS) in postmenopausal women for 12 and 24 months, the bone turnover markers osteocalcin (OC) and type I collagen C-terminal peptide (CTX) decreased significantly, despite the fact that there was no significant change in bone mineral density compared with the control group. Therefore, it indicates that prebiotics is important in regulating the intestinal flora and maintaining bone homeostasis. However, the regulatory effect of prebiotics is limited by the body’s tolerance. When prebiotics are excessive, the fermentation of undigested sugars and fibers in intestinal tract can cause flatulence and abdominal discomfort (Rizzoli, 2019).

### Fecal Microbiota Transplantation

FMT is a method to colonize the screened fecal suspension of healthy people in patients’ intestinal tract, which can change the composition of intestinal flora of the recipient directly and normalize it, so as to obtain therapeutic benefits (Wang et al., 2019). Studies have confirmed that FMT has a certain curative effect on infectious diseases, gastrointestinal diseases, nervous system diseases, liver diseases, and even cancers (Chen et al., 2019; Revolinski and Munoz-Price, 2019; Vaughn et al., 2019; Chauhan et al., 2020; Vendrik et al., 2020). Due to the direct effect on the intestinal flora, the regulation of bone homeostasis by FMT is possible. However, there is little research related to this field. Similarly, in order to implement personalized treatment, we need to consider such issues as host immune rejection, donor selection, and donor sample preparation in FMT.

## Conclusions and Future Perspectives

**Figure 6:**
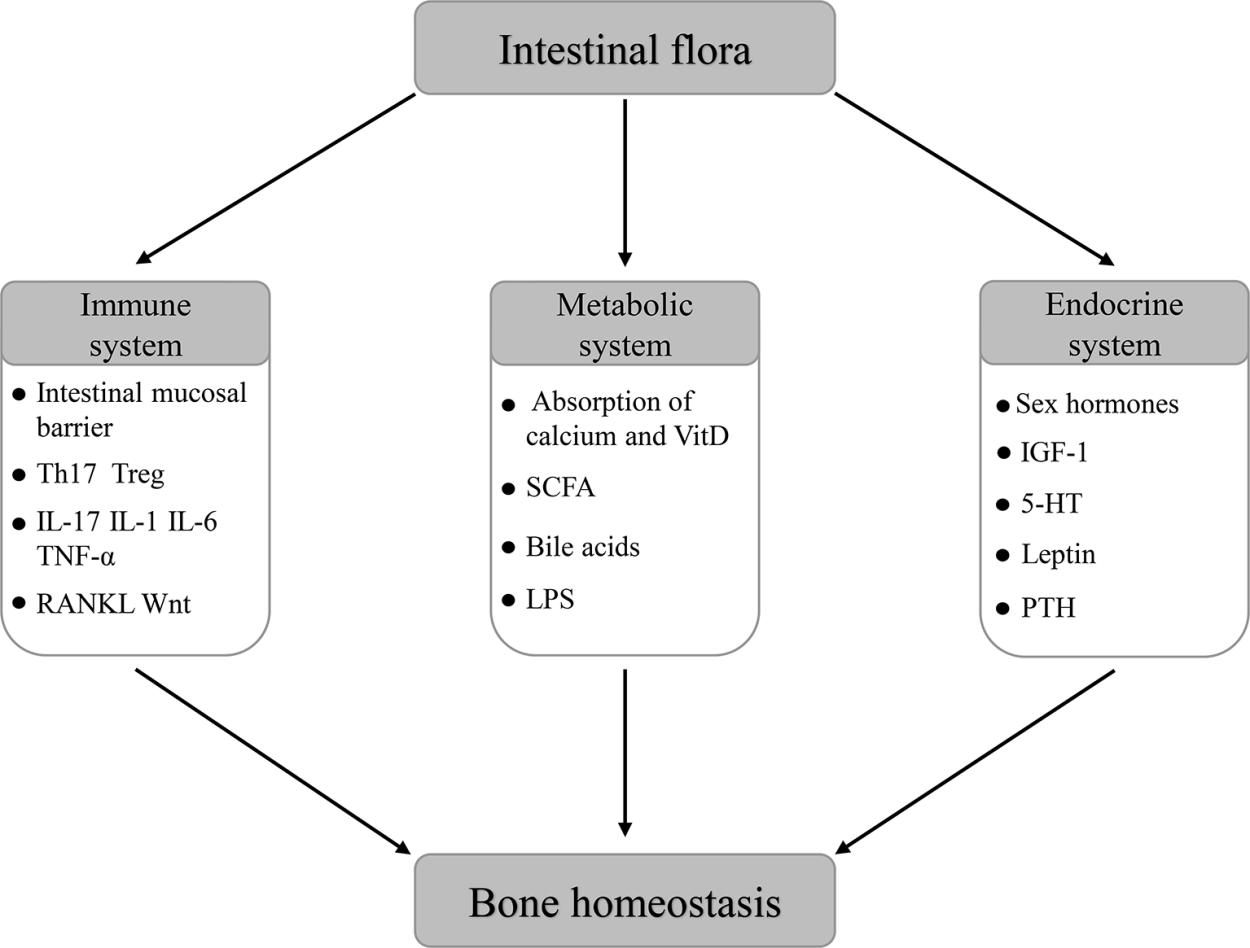
The role of intestinal flora in the regulation of bone homeostasis.

In summary, a large number of studies have preliminarily confirmed that the intestinal flora plays an important role in maintaining bone homeostasis by regulating the host immune, metabolic, and endocrine systems through different pathways, whenever in the state of growth or disease (**Figure 6**). However, current studies mainly focus on the effects of the intestinal flora on bone metabolic phenotype and bone turnover markers (BTMs). Studies on the specific molecular mechanism of their effects as well as the characterization and identification of important intestinal flora are further needed. At the same time, the directional change of the composition and function of host intestinal flora through the application of probiotics, prebiotics, and FMT provides a new target for the regulation of bone homeostasis. Nevertheless, the action mechanism, individual specificity, biosafety, and other problems still need to be further studied in the future so as to provide a more powerful theoretical basis and obtain more accurate efficacy for maintaining bone homeostasis through the target of the intestinal flora.

## Author Contributions

CL wrote the article. FL and GP designed and reviewed the paper. All authors contributed to the article and approved the submitted version.

## Funding

This work was supported by the National Natural Science Foundation of China under Grant 81802164; Henan Key R&D Promotion Project under Grant 22170108; Medical Scientific and Technological Research Project of Henan Province under Grant SBGJ2018029; Youth Innovation Fund Project of The First Affiliated Hospital of Zhengzhou University under Grant YNQN2017040.

## Conflict of Interest

The authors declare that the research was conducted in the absence of any commercial or financial relationships that could be construed as a potential conflict of interest.
